# Genome-Wide Association Analysis of Fresh Maize

**DOI:** 10.3390/ijms26157431

**Published:** 2025-08-01

**Authors:** Suying Guo, Rengui Zhao, Jinhao Lan

**Affiliations:** 1 College of Agronomy, Jilin Agricultural University, Changchun 130118, China; ggygsy@163.com; 2 College of Agronomy, Qingdao Agricultural University, Qingdao 266109, China

**Keywords:** fresh maize, SNPs, GWAS, candidate genes, gene function

## Abstract

This study measured eight key phenotypic traits across 259 fresh maize inbred lines, including plant height and spike length. A total of 82 single nucleotide polymorphisms (SNPs) significantly associated with these phenotypes were identified by applying a mixed linear model to calculate the best linear unbiased prediction (BLUP) values and integrating genome-wide genotypic data through genome-wide association analysis (GWAS). A further analysis of significant SNPs contributed to the identification of 63 candidate genes with functional annotations. Notably, 11 major candidate genes were identified from multi-trait association loci, all of which exhibited highly significant P-values (<0.0001) and explained between 7.21% and 12.78% of phenotypic variation. These 11 genes, located on chromosomes 1, 3, 4, 5, 6, and 9, were functionally involved in signaling, metabolic regulation, structural maintenance, and stress response, and are likely to play crucial roles in the growth and physiological processes of fresh maize inbred lines. The functional genes identified in this study have significant implications for the development of molecular markers, the optimization of breeding strategies, and the enhancement of quality in fresh maize.

## 1. Introduction

GWAS is a powerful tool for identifying the genetic variations associated with complex traits in natural populations. It leverages the principle of linkage disequilibrium (LD) to systematically analyze large-scale genotypic and phenotypic data, enabling the precise localization of loci influencing target traits. Two key parameters frequently used to characterize LD are r^2^ and D’, which capture distinct aspects of gene linkage. Specifically, r^2^ reflects the combined effects of mutation and recombination, and D’ emphasizes historical recombination events [1]. Among both parameters, r^2^ is considered more objective in assessing the degree to which markers are associated with quantitative trait loci (QTL) and is widely employed as an index for measuring LD between molecular markers in GWAS.

Both D’ and r^2^ range from 0 to 1, with higher values indicating stronger LD between allelic loci [2,3,4]. The overall level of LD within a genome is commonly characterized by the LD decay distance, which denotes the physical distance over which LD declines below a given threshold. This parameter is critical in GWAS study design as it determines the marker density required for effective and accurate locus detection. Notably, LD decay distances vary markedly across species. For instance, *Arabidopsis thaliana* exhibits an LD decay distance of approximately 250 kb, wheat up to 5 Mb [5,6], and *Drosophila* less than 1 kb [7]. Significant differences in LD decay were also observed among subpopulations within a single species. In maize, elite inbred lines exhibit an LD decay distance of approximately 100 kb, whereas landraces and genetically diverse inbred lines show much shorter decay distances of approximately 1–1.5 kb [8,9,10].

Wang et al. (2023) [11] conducted GWAS on i-traits using 228 maize self-crosses, identifying 4945 SNPs, 2603 genetic loci, and 1974 corresponding candidate genes associated with agronomic traits. Through functional validation via gene mutation analysis, they confirmed that the candidate gene *ZmVATE* regulated plant height-related traits in maize. Che et al. (2024) [12] measured the plant height across 132 wheat varieties under four distinct environmental conditions. Utilizing the wheat 35 K SNP microarray and a mixed linear model (MLM), they performed GWAS and demonstrated that plant height variation was significantly influenced by environmental factors. A total of 22 SNP loci were significantly associated with plant height, of which 7 were repeatedly detected under two to three environmental conditions and were classified as stable association loci. A homology-based functional analysis of 265 genes located within 1 Mb upstream and downstream of these loci led to the identification of seven candidate genes potentially involved in plant height development.

Qian et al. (2024) [13] constructed an association panel of 580 maize inbred lines with broad genetic diversity and used 31,826 genome-wide SNP markers to perform GWAS on plant height and ear height, based on three years of phenotypic data and BLUE. They identified 58 significant SNPs under four environmental conditions, including 6 co-localized loci, 3 associated with plant height, and 4 with ear height. Within a 440 kb window flanking the co-localized markers, 76 genes were identified, of which 66 had known functions. Gene annotation and GO enrichment analyses highlighted seven candidate genes with strong potential roles in controlling plant and ear height, including *Zm00001d042611*, which encodes GA20ox3, a gene previously confirmed to regulate plant height in maize. Zhou et al. (2016) [14] employed the Tuck 478 × Qi 319 hybrid population and detected 14 QTLs associated with the plant height and another 14 with the ear height. After further refinement, they identified two major candidate genes, *GRMZM2G325907* and *GRMZM2G108892*, with significant effects on both traits. Paciorek et al. (2022) [15] reported that reduced expression levels of *ZmGA20ox3* and *ZmGA20ox5* resulted in a decreased plant height in maize.

Despite the rapid development of fresh maize, its popularity and research attention remain limited compared with common maize, and GWAS on its genetic basis are still relatively scarce. In this study, phenotypic characterization was combined with genome-wide SNP markers to perform GWAS with the objective of identifying loci significantly associated with yield-related traits in fresh maize and screening the corresponding candidate genes.

## 2. Results

### 2.1. LD Analysis in the Fresh Maize Inbred Lines

**Figure 1:**
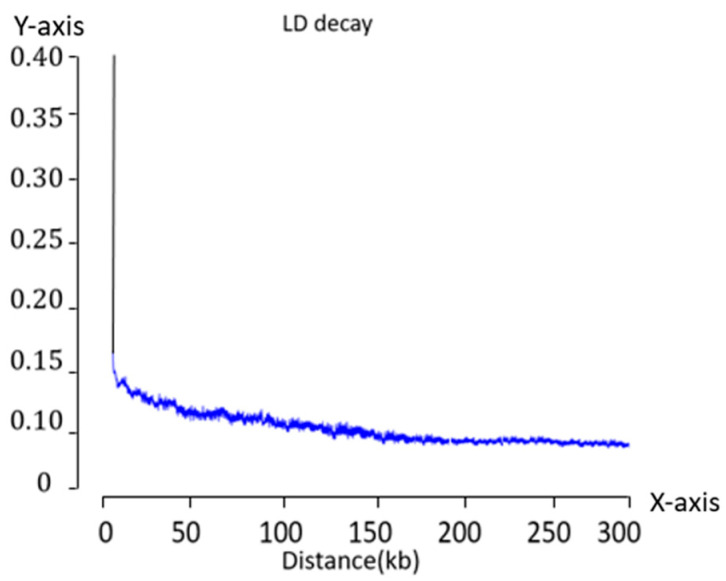
Fresh maize inbred LD attenuation map. *X*-axis: physical distance between SNP pairs (kb). *Y*-axis: LD strength measured by r^2^.

The average LD decay distance of the association population was calculated using PopLDdecay v3.43 software. The LD analysis of the maize inbred population revealed that the r^2^ value declined rapidly with increasing physical distance between loci. When an r^2^ threshold of 0.15 was applied as the intercept, the average LD decay distance across all chromosomes was approximately 3 kb. Moreover, the rapid decline in the LD coefficient suggested that the maize population possessed a relatively high genetic diversity. The results are shown in Figure 1.

### 2.2. GWAS of Traits Associated with Fresh Maize Inbred Lines

**Figure 2:**
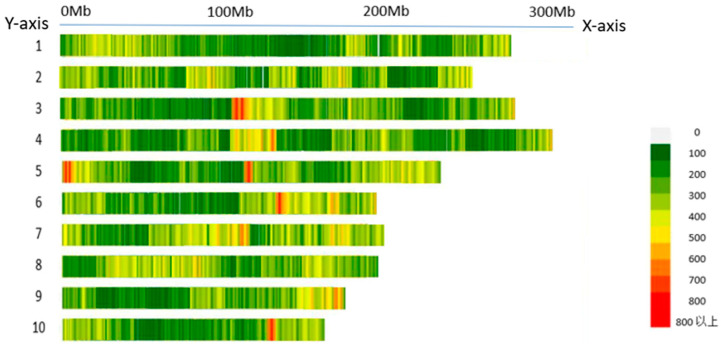
Distribution of SNPs on chromosomes in fresh maize. *X*-axis: physical position along chromosomes (0–300 Mb). *Y*-axis: chromosomes 1–10 of fresh maize. The color transition from colorless to red indicates an increase in the number of SNPs from 0 to above 800 (meaning of “above” in Chinese).

SNP screening was performed using the PLINK 2.0 software, with a minimum MAF threshold of no less than 0.05 and a missing data rate exceeding 10%, resulting in the identification of 225,863 high-quality SNP loci. As shown in Figure 2, these SNPs were evenly distributed across 10 maize chromosome pairs. Subsequently, the BLUE values for each trait were calculated using R, and GWAS was conducted for the eight phenotypic traits using MLM.

#### 2.2.1. Correlation Analysis with Ear Height

**Figure 3:**
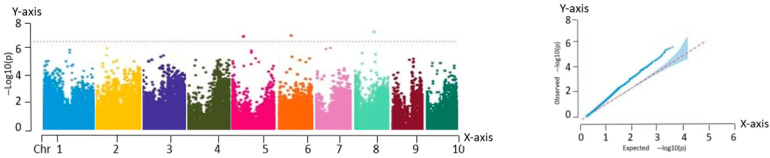
Manhattan and QQ plots obtained from GWAS of ear height. Manhattan plot—*X*-axis: genomic position across 10 maize chromosomes (Chr 1–10). *Y*-axis: −log_10_(P) value of SNP trait associations. QQ plot—*X*-axis: expected −log_10_(P). *Y*-axis: observed −log_10_(P).

As shown in the QQ plot (Figure 3), the predicted and observed values overlapped closely in the region corresponding to the loci of low significance, confirming the suitability of the selected model. The deviations observed in the upper tail, where the observed values exceeded the expected values, could reflect the true genetic effects associated with SNP variation.

The Manhattan plot in Figure 3 reveals three significant association loci located at chr5:214116269, chr6:4674859, and chr8:79284214. These loci may influence the yield by modulating ear height during the growth and development of fresh maize. Since ear height is closely related to traits such as lodging resistance and photosynthetic efficiency, it indirectly affects overall crop yield.

#### 2.2.2. Correlation Analysis with the Kernel Row Number

**Figure 4:**
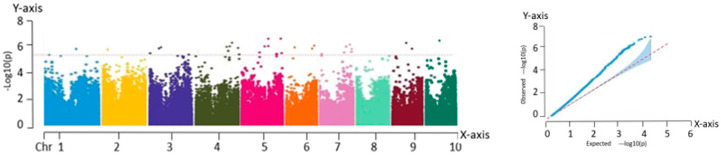
Manhattan and QQ plots obtained from GWAS of kernel row numbers. Manhattan plot—*X*-axis: genomic position across 10 maize chromosomes (Chr 1–10). *Y*-axis: −log_10_(P) value of SNP trait associations. QQ plot—*X*-axis: expected −log_10_(P). *Y*-axis: observed −log_10_(P).

The analysis of the Manhattan plot led to the identification of 31 significant association loci, located at the following genomic positions: chr1:37715366, chr2:5836129, chr3:3431342, chr3:208613443, chr3:210714590, chr3:210231278, chr4:1225341, chr4:182575530, chr4:185648982, chr4:185871044, chr4:182635028, chr4:184858702, chr5:94944612, chr5:182580617, chr5:182909034, chr5:182580617, chr5:88793905, chr5:94944612, chr5:209867042, chr6:102752903, chr6:110577129, chr6:166824260, chr7:1224023, chr7:124867427, chr7:125976199, chr7:128646920, chr7:124867427, chr7:125976199, chr9:26657512, chr9:134610897, and chr10:110297615. Among the traits analyzed, kernel row number is particularly important because of its strong correlation with yield. Therefore, it is essential to further investigate the genetic mechanisms underlying the yield formation in fresh maize. The results are shown in Figure 4.

#### 2.2.3. Correlation Analysis with Ear Length

**Figure 5:**
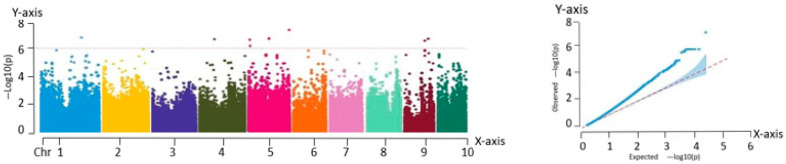
Manhattan and QQ plots obtained from GWAS of ear length. Manhattan plot—*X*-axis: genomic position across 10 maize chromosomes (Chr 1–10). *Y*-axis: −log_10_(P) value of SNP trait associations. QQ plot—*X*-axis: expected −log_10_(P). *Y*-axis: observed −log_10_(P).

As shown in Figure 5, eight significant association loci were identified based on the Manhattan plot. These loci are located at the following genomic positions: chr1:98163999, chr4:830558, chr5:94944612, chr5:182584005, chr5:182583809, chr5:183126231, chr9:25816452, and chr9:6657512. These loci may influence yield by regulating ear length, a key agronomic trait, during the growth and development of fresh maize.

#### 2.2.4. Correlation Analysis with the Kernels per Row

**Figure 6:**
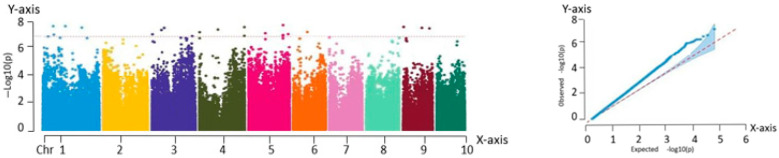
Manhattan and QQ plots obtained from GWAS of kernels per row. Manhattan plot—*X*-axis: genomic position across 10 maize chromosomes (Chr 1–10). *Y*-axis: −log_10_(P) value of SNP trait associations. QQ plot—*X*-axis: expected −log_10_(P). *Y*-axis: observed −log_10_(P).

A total of 20 significant association loci were identified based on the Manhattan plot, located at the following genomic positions: chr1:28609672, chr1:58717344, chr1:98340758, chr1:98163998, chr3:20861344, chr3:210714590, chr3:210231278, chr3:3431342, chr4:1225341, chr4:182635028, chr4:171730978, chr4:184858702, chr5:94944612, chr5:182909034, chr5:182192012, chr5:182580617, chr6:110577129, chr9:2712197, chr9:25847449, and chr9:134610897. The trait “kernels per row” was directly associated with maize grain yield, and the results suggested that these loci may influence yield by regulating the kernel number per row in fresh maize. The findings are shown in Figure 6.

#### 2.2.5. Correlation Analysis with Pollen-Shedding Period

**Figure 7:**
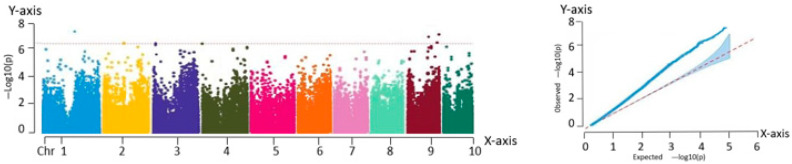
Manhattan and QQ plots obtained from GWAS during the pollen-shedding period. Manhattan plot—*X*-axis: genomic position across 10 maize chromosomes (Chr 1–10). *Y*-axis: −log_10_(P) value of SNP trait associations. QQ plot—*X*-axis: expected −log_10_(P). *Y*-axis: observed −log_10_(P).

As shown in Figure 7, four significant association loci were identified using the Manhattan plot. These loci are located at the following genomic positions: chr1:98163999, chr9:99366037, chr9:110251975, and chr9:130883359. The pollen-shedding period is a critical factor influencing pollination and fertilization in maize and plays a vital role in kernel formation. The identification of these association loci provides an important foundation for the further investigation of the factors affecting the yield of fresh maize and offers valuable insights into its genetic improvement.

#### 2.2.6. Correlation Analysis with Silking Stage

**Figure 8:**
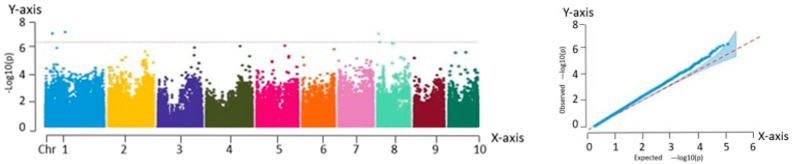
Manhattan and QQ plots obtained from GWAS during the days to silking stage. Manhattan plot—*X*-axis: genomic position across 10 maize chromosomes (Chr 1–10). *Y*-axis: −log_10_(P) value of SNP trait associations. QQ plot—*X*-axis: expected −log_10_(P). *Y*-axis: observed −log_10_(P).

Three significant association loci were identified based on the Manhattan plot (Figure 8). These loci are located at chr1:98163998, chr1:98163999, and chr8:79284214. It is highly likely that these loci influenced yield by regulating the silking stage, a key developmental trait in the growth of fresh maize.

#### 2.2.7. Correlation Analysis with Ear Diameter

**Figure 9:**
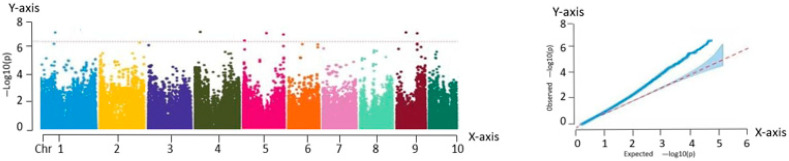
Manhattan and QQ plots obtained from GWAS of ear diameter. Manhattan plot—*X*-axis: Genomic position across 10 maize chromosomes (Chr 1–10). *Y*-axis: −log_10_(P) value of SNP trait associations. QQ plot—*X*-axis: Expected −log_10_(P). *Y*-axis: Observed −log_10_(P).

Seven association loci were identified using a Manhattan plot (Figure 9). These loci were located at chr1:98163999, chr4:830558, chr5:182909034, chr5:183126164, chr5:183126231, and chr9:25847449. Ear diameter, which is closely associated with kernel number and volume, represents a critical trait that influences the yield of fresh maize. These loci could contribute to yield variation by regulating ear diameter during plant development.

#### 2.2.8. Correlation Analysis with Plant Height

**Figure 10:**
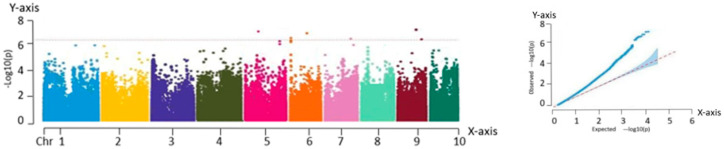
Manhattan and QQ plots obtained from GWAS of plant height. Manhattan plot—*X*-axis: Genomic position across 10 maize chromosomes (Chr 1–10). *Y*-axis: −log_10_(P) value of SNP trait associations. QQ plot—*X*-axis: Expected −log_10_(P). *Y*-axis: Observed −log_10_(P).

As shown in Figure 10, six association loci were identified in the Manhattan plot. These loci were located at chr5:182192012, chr6:4674859, chr6:108255064, chr7:152064165, chr9:25847449, and chr9:2712197. Plant height is a key agronomic trait that influences lodging resistance and photosynthetic efficiency in maize. These results suggest that these loci may affect yield by regulating plant height in fresh maize.

Overall, a total of 82 co-localized SNPs were identified through the analysis. Among these, three loci associated with ear height were located on chromosomes 5, 6, and 8, whereas six loci associated with plant height were distributed across chromosomes 5, 6, 7, and 9. A shared marker, 6:4674859 on chromosome 6, was co-localized for both plant height and ear height, suggesting its potential role as a key SNP regulating both traits. Additionally, 31 loci associated with kernel rows per ear were identified on chromosomes 1, 2, 3, 4, 5, 6, 7, 9, and 10, and 20 loci associated with kernels per row were mapped to chromosomes 1, 3, 4, 5, 6, and 9. Seven loci were co-localized between the two traits (kernel rows per ear and kernels per row), including 3:3431342 on chromosome 3; 4:1225341, 4:182635028, and 4:184858702 on chromosome 4; 5:182909034 on chromosome 5; 6:110577129 on chromosome 6; and 9:134610897 on chromosome 9. These shared loci may represent key genomic regions that influence yield-related traits in fresh maize. These markers may represent key SNPs that regulate both kernel rows per ear and kernels per row. Among the identified co-localized loci, several were associated with different developmental stages. Markers related to the pollen-shedding period were located on chromosomes 1 and 9, while three loci associated with the silking period were distributed on chromosomes 1 and 8. Notably, marker 1:98163999 on chromosome 1 was co-localized for both pollen-shedding and silking traits, suggesting that it may be a critical SNP that simultaneously regulates these reproductive stages. Additionally, eight markers were associated with ear length, and seven were associated with ear diameter, distributed across chromosomes 1, 4, 5, and 9. Four SNPs, such as 1:98163999 (chr1), 4:830558 (chr4), 5:183126231 (chr5), and 9:25847449 (chr9), were co-localized between ear length and ear diameter, indicating their potential roles in controlling the ear morphology. Overall, 12 SNPs were repeatedly identified across multiple traits, with 1:98163999 appearing in four traits: pollen-shedding period, silking period, ear length, and ear diameter. These results suggest that these loci, particularly 1:98163999, may harbor conserved functional genes involved in key developmental and yield-related processes in fresh maize.

### 2.3. Candidate Gene Analysis

LD decay analysis was conducted on the association population. When the r^2^ threshold was set to 0.15, the average LD decay distance across all chromosomes was approximately 3 kb. Based on the physical locations of the significantly associated SNPs, candidate genes were identified within the upstream and downstream LD intervals, using the average decay distance as the window. Functional annotation of the screened genes was performed using protein sequence alignment.

#### 2.3.1. Candidate Genes Associated with Ear Height

Ear height is a critical trait that influences the crop lodging resistance and photosynthetic efficiency of the plant population. In this study, three genetic loci that were significantly associated with ear height were identified using GWAS. A 3 kb genomic region upstream and downstream of each locus was thoroughly examined, leading to the identification of three candidate genes. The detailed information is provided in Table 1.

#### 2.3.2. Candidate Genes Associated with the Kernel Row Number

Kernel row number is a key agronomic trait that directly influences maize yield. GWAS identified 31 loci significantly associated with this trait. A genomic region, approximately 3 kb upstream and downstream of each locus, was examined to screen for candidate genes. The functional annotation of the identified genes resulted in the selection of 22 high-confidence candidate genes. The details are provided in Table 2.

#### 2.3.3. Candidate Genes Related to Ear Length

Ear length serves as a major indicator of the developmental potential of female inflorescences in maize. The association analysis of this trait identified eight significant loci. Further gene mining within approximately 3 kb upstream and downstream of these loci led to the identification of five candidate genes. The candidate genes are listed in Table 3.

#### 2.3.4. Candidate Genes Associated with Kernels per Row

The correlation analysis of kernels per row identified 20 significant loci. Candidate gene mining was conducted within approximately 3 kb upstream and downstream of these loci, resulting in the identification of 18 candidate genes. Among these, 16 genes were found to have known functional annotations (Table 4).

#### 2.3.5. Candidate Genes Associated with Pollen-Shedding Period

The association analysis of pollen-shedding period traits identified four significant loci. Candidate gene mining was performed within approximately 3 kb of these loci, leading to the identification of three candidate genes with functional annotations (Table 5).

#### 2.3.6. Candidate Genes Associated with Silking Stage

The correlation analysis of silking stage traits identified three significant loci. Candidate genes were screened within approximately 3 kb of each locus and functionally annotated, resulting in the identification of two candidate genes (Table 6).

#### 2.3.7. Candidate Genes Associated with Ear Diameter

The analysis of the ear diameter trait identified seven significant loci associated with yield through GWAS. Candidate genes within approximately 3 kb of each locus were screened and functionally annotated, resulting in the identification of seven candidate genes, six of which had known functional annotations (Table 7).

#### 2.3.8. Candidate Genes Associated with Plant Height

The GWAS of plant height traits identified six significant loci associated with yield. Candidate genes located within approximately 3 kb of each locus were screened and functionally annotated, resulting in the identification of six candidate genes, all of which had known functional annotations (Table 8).

Based on the above analysis, the B73_RefGen_v5 reference genome was utilized for comparative analysis using a 3 kb LD decay threshold (r^2^ = 0.15) and co-localized SNPs identified in the population. Candidate genes located within 3 kb upstream and downstream of the significant markers were screened, resulting in the detection of 71 genes. Functional annotation was conducted using MaizeGDB and NCBI databases, and 63 of these genes were successfully annotated.

#### 2.3.9. Major Candidate Genes

Based on the GWAS of eight traits, including ear height, kernel rows per ear, ear length, kernels per row, pollen-shedding period, silking period, ear diameter, and plant height, 11 major candidate genes were identified by screening the loci appearing repeatedly across multiple traits. These genes included *GRMZM2G174650*, *GRMZM2G400929*, *GRMZM2G073823*, *GRMZM2G176437*, *GRMZM2G060470*, *GRMZM2G106680*, *GRMZM2G007466*, *GRMZM2G563728*, *GRMZM2G134888*, *GRMZM2G066784*, and *GRMZM2G042080* (Table 9).

#### 2.3.10. Correlation Analysis of the 11 Candidate Genes Screened

In this study, a correlation analysis was performed between the 11 identified candidate genes and phenotypic traits. The results indicated that *GRMZM2G174650* was significantly associated with pollen-shedding period, silking period, ear length, and ear diameter, implying its potential pivotal role in reproductive development and ear formation in fresh maize.

The five genes *GRMZM2G400929*, *GRMZM2G176437*, *GRMZM2G060470*, *GRMZM2G134888*, and *GRMZM2G066784* were all associated with kernel row number and kernels per row, indicating their potential involvement in regulating grain number-related developmental processes in fresh maize.

*GRMZM2G073823* and *GRMZM2G007466* were significantly associated with ear length and diameter, suggesting their potential role in regulating ear morphology in fresh maize. *GRMZM2G106680* exhibited associations with kernel row number, kernels per row, and ear diameter, indicating a broader regulatory influence on ear development. Moreover, *GRMZM2G042080* was associated with ear length, kernels per row, ear diameter, and plant height, implying its involvement in multiple traits related to both plant architecture and ear development in fresh maize (Table 10).

#### 2.3.11. Statistics Related to Different Loci of the 11 Candidate Genes Screened

The 11 candidate genes identified were further examined (Table 11). All genes exhibited P-values that satisfied the study’s significance threshold, with phenotypic contributions reaching 12.78%, which could strongly support their critical associations with key agronomic traits.

The GWAS of eight traits, such as ear height and kernel row number, successfully revealed 11 major candidate genes distributed across chromosomes 1, 3, 4, 5, 6, and 9. The functional annotation revealed that *GRMZM2G174650*, a member of the VQ motif-containing protein family, may be involved in plant hormone signal transduction, *GRMZM2G400929* encodes a carboxylesterase implicated in lipid metabolism, and *GRMZM2G073823* contains a GRAS domain that could function in gibberellin signaling and plant growth regulation. As a Harbinger transposase-derived protein, *GRMZM2G176437* may contribute to genomic stability by modulating the transposon activity. *GRMZM2G060470* encodes a nucleotide-diphospho-sugar transferase that is potentially involved in glycosylation modification and cell wall biosynthesis, whereas *GRMZM2G106680* may regulate intracellular ion homeostasis. *GRMZM2G007466*, a protein tyrosine kinase, is associated with cellular signal transduction. *GRMZM2G563728* encoded a VPS13 vacuolar protein sorting-associated protein, possibly essential for endomembrane system functionality. *GRMZM2G134888* is an amino acid transporter that can influence nitrogen metabolism. *GRMZM2G066784*, encoding an 11-oxo-β-amyrin 30-oxidase, may participate in phytosterol and triterpenoid biosynthesis, and *GRMZM2G042080*, as a superoxide dismutase, may play a critical role in antioxidant stress responses. These findings establish a molecular basis for the further exploration of the genetic mechanisms that regulate important traits in fresh maize.

## 3. Discussion

Ear and grain traits in maize are regulated by complex gene networks and are highly susceptible to environmental influences [16]. In a study by Yang et al. (2023) [17], GWAS was conducted using 201 maize inbred lines, identifying 21 stably associated SNP loci across traits, such as plant height, ear height, and number of tassel branches, specifically, 10 SNPs for plant height, 5 for ear height, and 6 for tassel branch number. Similarly, Qian et al. (2024) [13] analyzed 580 genetically diverse inbred lines and identified 58 significant SNPs across four environments, including 6 co-localized loci, with 3 associated with plant height and 4 with ear height. Wang et al. (2016) [18] reported 206 trait-associated SNPs from a GWAS on 201 inbred lines. In this study, a GWAS of 259 genetically distinct fresh maize inbred lines identified candidate genes involved in flowering traits (e.g., pollen-shedding and silking), plant architecture, and yield components. GWAS remains an indispensable tool for dissecting the genetic architecture of complex traits and elucidating the influence of genotypic variation on phenotypes. The targeted mining of functional SNPs related to kernel row number and kernel count per row, and further candidate gene validation, laid a theoretical foundation for advancing the genetic understanding of maize yield-related traits.

Qi et al. (2025) [19] identified 13 SNP loci significantly associated with flowering-stage traits in the GWAS conducted over two consecutive years. These loci were distributed across chromosomes 1, 2, 3, 4, 5, 8, and 9 in maize. Notably, marker 1-19655896 on chromosome 1 was consistently detected at two critical flowering stages, including tassel emergence and pollen dispersal, which suggested its potential role in reproductive timing. Based on these associations, 18 candidate genes were further screened, 10 of which were successfully annotated. Specifically, *Zm00001d032633* encodes the phytoalexin N-methyltransferase, *Zm00001d043842* encodes a GPI-anchoring protein, *Zm00001d013415* encodes the receptor-like protein kinase TMK, and *Zm00001d010489* encodes the calmodulin-binding protein, all of which may participate in the signaling and developmental processes relevant to maize flowering. The identification of these potential candidate genes offers a robust theoretical foundation for breeding new maize varieties with optimized flowering periods and can be expected to drive significant progress in varietal selection and improvement. In maize, the pollen dispersal period corresponded to the stage when the male tassel released pollen, whereas the silking period referred to the emergence of female silk from the husk to receive pollen. Precise synchronization between these two stages is critical for successful pollination and fertilization. Successful fertilization and subsequent seed formation can only occur when pollen dispersal and silk emergence are precisely synchronized. If the two processes are not synchronized, particularly earlier dispersal than silk emergence, pollen may lose its viability. On the other hand, if silk emergence occurs before pollen dispersal, the silks may become excessively elongated and prone to interference with pollination, which can result in a reduced fruit set rate and ultimately affect corn yield. The identification of functional SNPs associated with pollen dispersal and silk emergence, along with the functional validation of candidate genes, could provide a theoretical foundation for genetic research on fresh maize.

According to Ma et al. (2023) [20], a total of 11 SNP loci associated with maize ear length were identified using seven multi-environment genome-wide association study (MGWAS) methods. These loci were located on chromosomes 1, 2, 5, 6, 7, 8, and 10. Zhao et al. (2019) [21] employed the composite interval mapping (CIM) method and detected 62 loci associated with spikelet traits under a single environmental condition, as well as 38 loci under drought stress. Both ear length and diameter were strongly correlated with maize yield. Generally, longer ears and thicker ear diameters correspond to increased ear volume, which provides more space for grain development, thereby enhancing the number of kernels and kernel weight per ear and improving the overall yield potential. Studies focusing on ear length and diameter can elucidate the mechanisms underlying yield formation in various maize genotypes and offer a theoretical foundation for high-yield breeding. The identification of functional SNPs related to these traits, along with the subsequent functional validation of candidate genes, can contribute significantly to the genetic improvement and yield enhancement of fresh maize.

Candidate genes refer to the genes localized in specific chromosomal regions that are hypothesized to influence phenotypic traits. Although the functional expression of many candidate genes remains unclear, ongoing studies have focused on their protein products [22]. These genes may function as structural, regulatory, or metabolic pathway-related genes that affect phenotypic development [23]. Numerous genes have been shown to be closely associated with traits such as plant height, ear height, and number of tassel branches in maize, playing significant roles in these developmental processes [24,25,26,27,28,29,30,31,32,33,34,35,36,37,38,39,40]. These genes are typically involved in hormone biosynthesis, signal transduction, cellular division and growth, transcriptional regulation, and metabolism. Based on the analysis of LD decay distance, each SNP locus was used as a reference point, and a 3 kb region upstream and downstream of its physical position was defined as the candidate gene search region. Using this query range, a search was conducted for protein-coding genes in the maize B73 genome (version V5) using MaizeGDB, and 63 candidate genes were identified. These functional genes are mainly involved in the regulation of plant growth, material metabolism, or environmental adaptation. Their roles include binding to the promoter regions of target genes to regulate gene expression; participating in the signal transduction, localization, and functional regulation of membrane proteins; involvement in nitrogen metabolism, nutrient partitioning, and utilization in plants; the regulation of abscisic acid levels; the modulation of stress-responsive gene expression to enhance stress resistance; and functioning as transcription factors in regulating the expression of genes related to biosynthetic processes.

## 4. Materials and Methods

### 4.1. Materials

A total of 259 fresh maize inbred lines with desirable agronomic traits were collected from various regions across China and used as the experimental population. Field trials were conducted at two locations over two consecutive years. Eight phenotypic traits, such as ear height, kernel row number, ear length, kernels per row, pollen-shedding period, silking stage, ear diameter, and plant height, were recorded for all 259 lines.

In 2023, 259 fresh maize samples were cultivated from June to September at Mizhou Seed Industry, Zhucheng, Weifang, Shandong Province, China (longitude 119°0′–119°43′ E, latitude 35°42′–36°21′ N). The average temperature during this period was approximately 25 °C, with the total precipitation reaching 500 mm. In 2024, the same 259 fresh maize samples were cultivated from June to September at the same location. During this period in 2024, the average temperature was 26 °C and the precipitation totaled 490 mm.

Parallel trials were conducted at the Pingdu experimental site in Qingdao, Shandong Province (36°47′ N, 119°58′ E). The average temperature during the period of June to September in 2023 was approximately 25 °C, accompanied by 470 mm of precipitation. In 2024, the same experimental design was repeated at the same site, the same period in 2024 recorded an average temperature of 26 °C and a precipitation level of 450 mm.

### 4.2. Methods

#### 4.2.1. Whole Genome Sequencing and SNP Marker Development

Population genotyping was performed using targeted sequencing-based genotyping and targeted sequencing (GBTS). The resulting SNP data were further filtered using the PLINK software, with a minimum allele frequency (MAF) threshold of ≥0.05 and a missing data rate cutoff of ≤10%. After quality control, 225,863 high-quality SNP loci were retained for subsequent analyses.

#### 4.2.2. GWAS Analytical and Phenotypic Statistical Methods

The filtered SNP loci were used as the genotypic data and combined with the phenotypic data of the inbred lines for GWAS using a mixed linear model (MLM) implemented in TASSEL 5.0. The significance thresholds for association were adjusted using the Bonferroni correction method (0.01/N and 0.05/N, where N represents the total number of SNP markers), and the corresponding threshold lines were determined based on the corrected P-values.

The descriptive statistical analysis of phenotypic traits, including mean, standard deviation, coefficient of variation, range, skewness, and kurtosis, was performed using Python 3.11.

#### 4.2.3. LD

The genome-wide LD among multiple polymorphic loci is commonly evaluated using the r^2^ statistic, which quantifies the degree of LD between two loci on a scale from 0 to 1. A value of 0 indicates no LD, whereas a value of 1 represents complete LD. This value reflects the deviation between the observed co-occurrence frequency of the two alleles and the expected frequency under random association. Based on the genotype distributions within a population, an LD decay curve can be constructed in which r^2^ can be used to visualize the extent and decline of LD across physical distances.

#### 4.2.4. GWAS

Association analysis, also known as LD analysis, is a statistical approach for characterizing the relationship between genetic variants and phenotypic traits. This method enables the identification of genetic loci that are significantly associated with specific traits using statistical testing. Based on high-quality variant loci filtered using PLINK, a total of 225,863 SNP markers were retained. GWAS was performed for eight phenotypic traits across 259 fresh maize inbred lines using the MLM implemented in TASSEL. To visualize the association results, Manhattan plots were generated using the qqman package in R, showing the chromosomal distribution of the significant SNP loci. In parallel, QQ plots were constructed to evaluate the fit between observed and expected P-value distributions. In the QQ plot, the SNPs aligned along the diagonal in the lower left region indicate model consistency, while the SNPs deviating above the diagonal in the upper right corner represent loci with significantly stronger associations than expected.

#### 4.2.5. Candidate Gene Prediction and Functional Annotation

To interpret the results of LD decay distance, the physical interval corresponding to an r^2^ value of 0.15 was identified both upstream and downstream of each significant SNP locus. This interval was defined as the candidate region for gene identification. Using the MaizeGDB database (www.maizegdb.org/gbrowse) and incorporating information on the chromosomal location of each SNP, protein-coding genes within these regions were retrieved. All annotations were based on the B73 maize reference genome version V5, which ultimately resulted in the identification of candidate genes associated with the respective phenotypic traits.

## 5. Conclusions

The GWAS of 259 fresh maize inbred lines identified 82 significant SNPs, of which 75 were associated with plant architecture traits and 7 with flowering time characteristics. These loci were significantly correlated with key agronomic traits including anthesis date (pollen-shedding), silking date, and plant type attributes.

In this study, eight major phenotypic traits were evaluated across the 259 inbred lines. An MLM was employed to conduct GWAS, resulting in the identification of 82 trait-associated SNPs. From these, 63 candidate genes with functional annotations were screened, and 11 core candidate genes were identified using multi-trait association analysis. These 11 genes exhibited highly significant associations (*p* < 0.0001) and explained 7.21% to 12.78% of the phenotypic variation. They were distributed on chromosomes 1, 3, 4, 5, 6, and 9. Functional predictions indicated that these genes were likely involved in key biological processes, including phytohormone signaling, metabolic regulation, structural maintenance, and stress response pathways.

## Figures and Tables

**Table 1:** Candidate genes related to ear height.

Chr	Pos	Genetic Code	Functional Annotation
5	214116269	*GRMZM2G180422*	Transmembrane ProteinDDB_G0292058
6	4674859	*GRMZM2G563728*	Vacuolar Protein Sorting-Associated Protein VPS13
8	79284214	*GRMZM2G073888*	Purine-nucleoside phosphorylase/PNPase

**Table 2:** Candidate genes related to kernel row number.

Chr	Pos	Genetic Code	Functional Annotation
1	37715366	*GRMZM2G015281*	AP2 domain (AP2)
2	5836129	*GRMZM2G023921*	VQ motif-containing protein family
3	3431342	*GRMZM2G400929*	Carboxylesterase
3	208613443	*GRMZM2G077607*	NO
3	210714590	*AC230013.2_FG007*	Large subunit ribosomal protein L18Ae
3	210231278	*GRMZM2G058518*	NAC domain-containing protein73
4	1225341	*GRMZM2G176437*	Harbinger transposase-derived protein
4	182575530	*GRMZM2G439339*	Nucleoprotein TPR
4	185648982		
4	185871044	*GRMZM2G071288*	Peroxisomal membrane protein
4	182635028	*GRMZM2G141222*	NO
4	184858702	*GRMZM2G060470*	Nucleotide-diphospho-sugar transferases
5	94944612	*GRMZM2G109914*	S-acyltransferase
5	182580617		
5	182909034	*GRMZM2G106680*	LETM1 and EF-hand domain-containing protein1
5	182580617		
5	88793905	*GRMZM2G111136*	Transcription factor, SBP-box
5	94944612	*GRMZM2G109914*	
5	209867042	*GRMZM2G103128*	Galactoside 2-alpha-L-fucosyltransferase
6	102752903	*GRMZM2G042806*	ENT domain/Agenet domain
6	110577129	*GRMZM2G134888*	Amino acid transporter
6	166824260		
7	1224023	*GRMZM2G135808*	NO
7	124867427	*GRMZM2G067265*	Outer Armdynein light chain 1protein
7	125976199	*GRMZM2G065928*	ABA 8′-hydroxylase
7	128646920	*GRMZM2G082653*	E3 ubiquitin-protein ligase
7	124867427	*GRMZM2G067265*	Subgroup I amino transferase-related
7	125976199	*GRMZM2G065928*	ABA 8′-hydroxylase
9	26657512	*NO*	NO
9	134610897	*GRMZM2G066784*	11-oxo-beta-amyrin 30-oxidase
10	110297615	*GRMZM2G153456*	GLTSCR protein

**Table 3:** Candidate genes related to ear length.

Chr	Pos	Genetic Code	Functional Annotation
1	98163999	*GRMZM2G174650*	VQ motif-containing protein family
4	830558	*GRMZM2G073823*	GRAS domain family (GRAS)
5	94944612	*GRMZM2G109914*	S-acyl transferase
5	182584005		
5	182583809		
5	183126231	*GRMZM2G007466*	Protein-tyrosine kinase
9	25816452	*GRMZM2G042080*	Superoxide dismutase
9	6657512	*NO*	NO

**Table 4:** Candidate genes related to kernels per row.

Chr	Pos	Genetic Code	Functional Annotation
1	28609672	*GRMZM2G344388*	Mitogen-activated protein kinase
1	58717344	*GRMZM2G177532*	Mitochondrial metal transporter 1-related
1	98340758	*GRMZM2G001895*	F5D14.30 Protein-Related
1	98163998		
3	20861344	*GRMZM2G077607*	NO
3	210714590	*AC230013.2_FG007*	Large subunit ribosomal protein L18Ae
3	210231278	*GRMZM2G058518*	NAC domain containing protein73
3	3431342	*GRMZM2G400929*	Carboxylesterase
4	1225341	*GRMZM2G176437*	Harbinger transposase-derived protein
4	182635028	*GRMZM2G141222*	NO
4	171730978	*GRMZM2G099097*	Fatty acid hydroxylase
4	184858702	*GRMZM2G060470*	Nucleotide-di phospho-sugar transferases
5	94944612	*GRMZM2G109914*	S-acyltransferase
5	182909034	*GRMZM2G106680*	LETM1 and EF-hand domain-containing protein1
5	182192012	*GRMZM2G001033*	Nucleotide-sugar transporter
5	182580617		
6	110577129	*GRMZM2G134888*	Amino acid transporter
9	2712197	*GRMZM2G026833*	Myb-like DNA-binding domain
9	25816452	*GRMZM2G042080*	Superoxide dismutase
9	134610897	*GRMZM2G066784*	11-oxo-beta-amyrin30-oxidase

**Table 5:** Candidate genes related to the pollen-shedding period.

Chr	Pos	Genetic Code	Functional Annotation
1	98163999	*GRMZM2G174650*	VQ motif-containing protein family
9	99366037	*GRMZM2G033480*	NO
9	110251975	*GRMZM2G174444*	Monodehydroascorbate reductase (NADH)
9	130883359	*GRMZM2G015280*	Peroxidase

**Table 6:** Candidate genes related to the silking stage.

Chr	Pos	Genetic Code	Functional Annotation
1	98163998		
1	98163999	*GRMZM2G174650*	VQ motif-containing protein family
8	79284214	*GRMZM2G073888*	Purine-nucleoside phosphorylase/PNPase

**Table 7:** Candidate genes related to ear diameter.

Chr	Pos	Genetic Code	Functional Annotation
1	98163999	*GRMZM2G174650*	VQ motif-containing protein family
4	830558	*GRMZM2G073823*	GRAS domain family (GRAS)
5	182909034	*GRMZM2G106680*	LETM1 and EF-hand domain-containing protein1
5	209867042	*GRMZM2G103128*	Galactoside2-alpha-L-fucosyl transferase
5	183126231	*GRMZM2G007466*	Protein-tyrosine kinase
9	25816452	*GRMZM2G042080*	Superoxide dismutase
9	99366037	*GRMZM2G033480*	

**Table 8:** Candidate genes related to plant height.

Chr	Pos	Genetic Code	Functional Annotation
5	182192012	*GRMZM2G001033*	Nucleotide-sugar transporter
6	4674859	*GRMZM2G563728*	Vacuolar Protein Sorting-Associated Protein
6	108255064	*GRMZM2G701058*	Ethanolamine-phospho-transferase
7	152064165	*GRMZM2G134545*	Dof domain zinc finger (zf-Dof)
9	25816452	*GRMZM2G042080*	Superoxide dismutase
9	130883359	*GRMZM2G015280*	Peroxidase

**Table 9:** Main candidate genes.

Chr	Pos	Genetic Code	Functional Annotation
1	98163999	*GRMZM2G174650*	VQ motif-containing protein family
3	3431342	*GRMZM2G400929*	Carboxylesterase
4	830558	*GRMZM2G073823*	GRAS domain family (GRAS)
4	1225341	*GRMZM2G176437*	Harbinger transposase-derived protein
4	184858702	*GRMZM2G060470*	Nucleotide-diphospho-sugar transferases
5	182909034	*GRMZM2G106680*	LETM1 and EF-hand domain-containing protein1
5	183126231	*GRMZM2G007466*	Protein-tyrosine kinase
6	4674859	*GRMZM2G563728*	Vacuolar Protein Sorting-Associated Protein VPS13
6	110577129	*GRMZM2G134888*	Amino acid transporter
9	134610897	*GRMZM2G066784*	11-oxo-beta-amyrin 30-oxidase
9	25816452	*GRMZM2G042080*	Superoxide dismutase

**Table 10:** Main candidate genes and associated traits.

Chr	Position	Genetic Code	Related Traits	Occurrence Frequency of the Gene
1	98163999	*GRMZM2G174650*	pollen-shedding period, silking stage, ear length, ear diameter	4
3	3431342	*GRMZM2G400929*	kernel row number, kernels per row	2
4	830558	*GRMZM2G073823*	ear length, ear diameter	2
4	1225341	*GRMZM2G176437*	kernel row number, kernels per row	2
4	184858702	*GRMZM2G060470*	kernel row number, kernels per row	2
5	182909034	*GRMZM2G106680*	kernel row number, kernels per row, ear diameter	3
5	183126231	*GRMZM2G007466*	ear length, ear diameter	2
6	4674859	*GRMZM2G563728*	ear height, plant height	2
6	110577129	*GRMZM2G134888*	kernel row number, kernels per row	2
9	134610897	*GRMZM2G066784*	kernel row number, kernels per row	2
9	25847449	*GRMZM2G042080*	ear length, kernels per row, ear diameter, plant height	4

**Table 11:** Statistical data related to different loci of 11 candidate genes.

Marker	Chromosome	Position	*p*-Value	Phenotypic Variation Explained
1-98163999	1	98163999	6.65 × 10^−8^	9.56%
3-3431342	3	3431342	4.23 × 10^−6^	10.31%
4-830558	4	830558	5.23 × 10^−7^	11.26%
4-1225341	4	1225341	3.21 × 10^−6^	8.71%
4-184858702	4	184858702	3.72 × 10^−7^	7.39%
5-182909034	5	182909034	6.03 × 10^−6^	8.58%
5-183126231	5	183126231	2.34 × 10^−6^	8.23%
6-4674859	6	4674859	4.08 × 10^−7^	7.21%
6-110577129	6	110577129	3.00 × 10^−6^	9.38%
9-134610897	9	134610897	2.51 × 10^−8^	12.78%
9-25847449	9	25847449	4.82 × 10^−7^	9.03%

## Data Availability

Data available in a publicly accessible repository.
